# Navigating the complexity of atypical teratoid/rhabdoid tumor (ATRT) in pediatric neuro-oncology: Insights from clinical spectrum to therapeutic challenges

**DOI:** 10.1016/j.ijscr.2025.111354

**Published:** 2025-04-23

**Authors:** Ali Msheik, Mohamad Yazbeck, Abdulla Illeyan, Youssef Comair

**Affiliations:** a Neurological surgery, Hamad Medical Corporation, Qatar; b Neurosurgery, Clemenceau Medical Centre, Lebanon; c Professor of Neurosurgery, Johns Hopkins Hospital, MD, USA

**Keywords:** ATRT, Pediatric brain tumors, Infratentorial neoplasms, Neurosurgery, Chemotherapy, MRI findings

## Abstract

**Introduction and importance:**

Atypical teratoid/rhabdoid tumor (ATRT) is a rare and aggressive pediatric central nervous system malignancy, accounting for only 1–2 % of cases. Primarily affecting children under three years old, ATRT poses significant diagnostic and therapeutic challenges, with high recurrence rates and poor prognosis due to its rapid progression and lack of a standardized treatment protocol.

**Case presentation:**

We report the case of a 2-year-old male diagnosed with infratentorial ATRT after presenting with abnormal gait, vomiting, and ataxia following minor head trauma. Magnetic resonance imaging (MRI) revealed a mixed solid-cystic cerebellar lesion, prompting surgical resection. Despite postoperative chemotherapy, tumor progression was noted, leading to a second craniotomy, which achieved complete resection. Serial follow-up MRI until February 2025 showed no evidence of recurrence, and the patient remains symptom-free four years post-treatment.

**Conclusion:**

This case underscores the complexities of ATRT management, emphasizing the importance of early diagnosis, aggressive multimodal therapy, and vigilant radiological follow-up. While ATRT shares imaging similarities with medulloblastomas, its distinct histopathological features necessitate tailored treatment strategies. Maximal safe resection, followed by intensive chemotherapy, remains the cornerstone of treatment, highlighting the need for a multidisciplinary approach to improve long-term outcomes in pediatric ATRT patients.

## Highlights

• ATRT is highly malignant in kids <3 yrs, needing early, intensive treatment for better outcomes.
• Multimodal therapy (surgery + chemo) is vital, as shown in studies and our case.
• MRI shows typical ATRT signs: heterogeneous enhancement and solid-cystic lesions.
• Early aggressive treatment led to no recurrence and symptom-free status up to 2025.
• This case supports early diagnosis and multimodal therapy for improved prognosis.

## 1 Introduction

Atypical teratoid/rhabdoid tumor (ATRT) constitutes 1–2 % of pediatric central nervous system (CNS) neoplasms [1] and ranks as the predominant malignancy in children under 36 months at 17.3 % [1]. ATRT is highly aggressive. It is infratentorial in half of the reported cases [2]. A pediatric cohort reported a 34 % supratentorial incidence of ATRT [2]. While metastasis is observed in only 35.3 % of patients older than 6 months [2], 100 % of the patients under 6 months showed metastasis at diagnosis [3]. In adults, the sellar region and cerebral hemispheres are commonly affected. ATRTs histologically feature rhabdoid cells and mixed components of neuroectodermal, ectodermal, and mesenchymal cells. As per the World Health Organization (WHO) classification, embryonal tumors are designated as Grade 4 tumors [4] except CNS tumors with BCL6 corepressor internal tandem duplication and cribriform neuroepithelial tumors [4].

Cerebellar tumors manifest as headache, vomiting, gait abnormalities, and instability. The tumor appears typically hyperintense on T2-weighted magnetic resonance imaging (MRI), images, except for hemorrhagic regions, and exhibits heterogeneously enhancing T1 images with contrast [5]. Immunohistochemistry and genetic testing are for definitive diagnosis because the rhabdoid cells, which are characteristic, constitute a limited portion of the specimen. Up to 95 % of ATRTs display two inactive copies of the integrase interfactor-1 (INI-1) gene. Rhabdoid cells commonly express epithelial membrane antigen (EMA), vimentin, and smooth muscle actin (SMA) [6].

The prognosis is exceptionally poor due to recurrence at the primary tumor site or distant metastases in blood and cerebrospinal fluid (CSF) and the lack of standard treatment protocol for ATRTs. The mean survival despite surgical intervention, radiotherapy, and high-dose chemotherapy (HDCT) is 6–18 months [7]. Disease progression (PD) is observed in 77 % of cases, typically within a median of 160 days post-diagnosis [8]. Some authors suggest improved survival with the absence of metastasis at diagnosis, early adjuvant radiotherapy and HDCT, and diagnoses made after the year 2011 [8].

This report describes the case of a 2-year-old male child diagnosed with an infratentorial tumor, which underwent surgical intervention and was subsequently identified as an Atypical Teratoid Rhabdoid Tumor (ATRT).

## 2 Case presentation

**Fig. 1:**
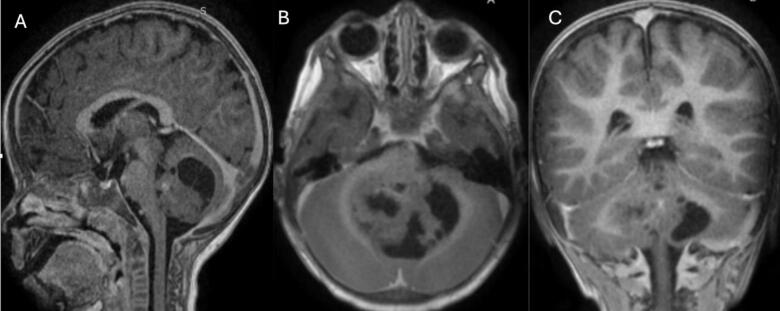
Magnetic resonance imaging of the brain: First MRI done before the first.

This is a case of a 3-year-old pediatric male patient born on December 13, 2018. At the age of 2, he manifested abnormal gait, vomiting, and ataxia, following a minor head trauma. An MRI examination showed a mixed solid cystic lesion measuring 56 × 37 × 36 mm within the cerebellum (Figs. 1 A, B, and C). Laboratory workup was unremarkable.

**Fig. 2:**
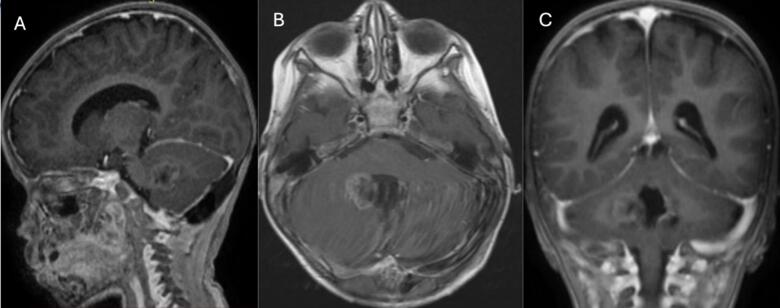
Magnetic resonance imaging of the brain: First MRI done after the first surgical intervention.

**Fig. 3:**
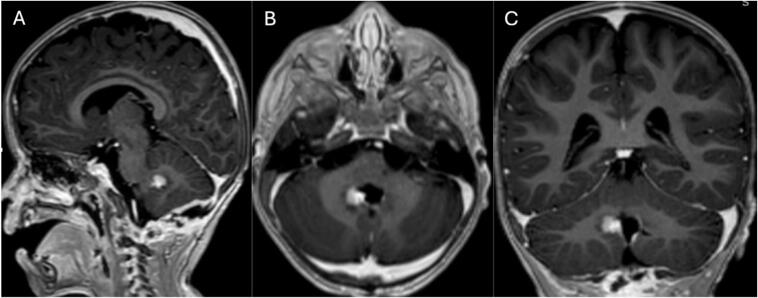
Magnetic resonance imaging of the brain: first imaging after chemotherapy initiation post-surgical intervention.

**Fig. 4:**
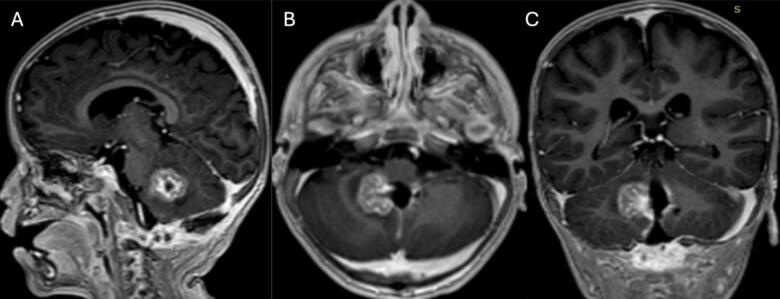
Magnetic resonance imaging of the brain: Second MRI after the First craniotomy showing an increase in the size of the enhancing residual lesion.

**Fig. 5:**
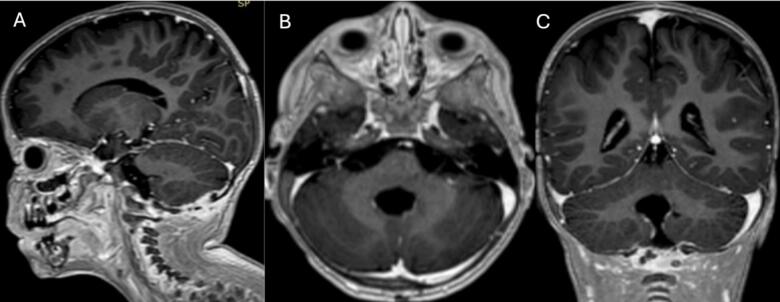
Magnetic resonance imaging of the brain: First imaging after the second craniotomy showing complete resection.

**Fig. 6:**
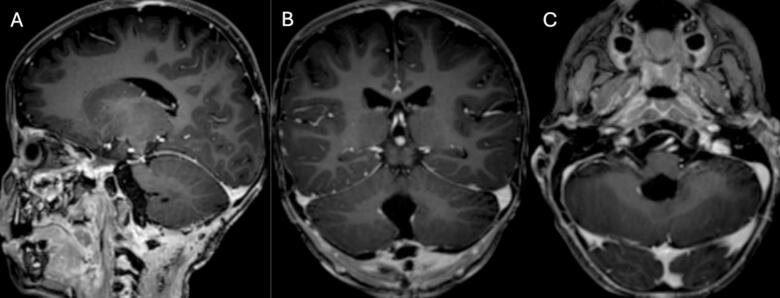
Magnetic resonance imaging of the brain: Second follow-up imaging showing total remission.

**Fig. 7:**
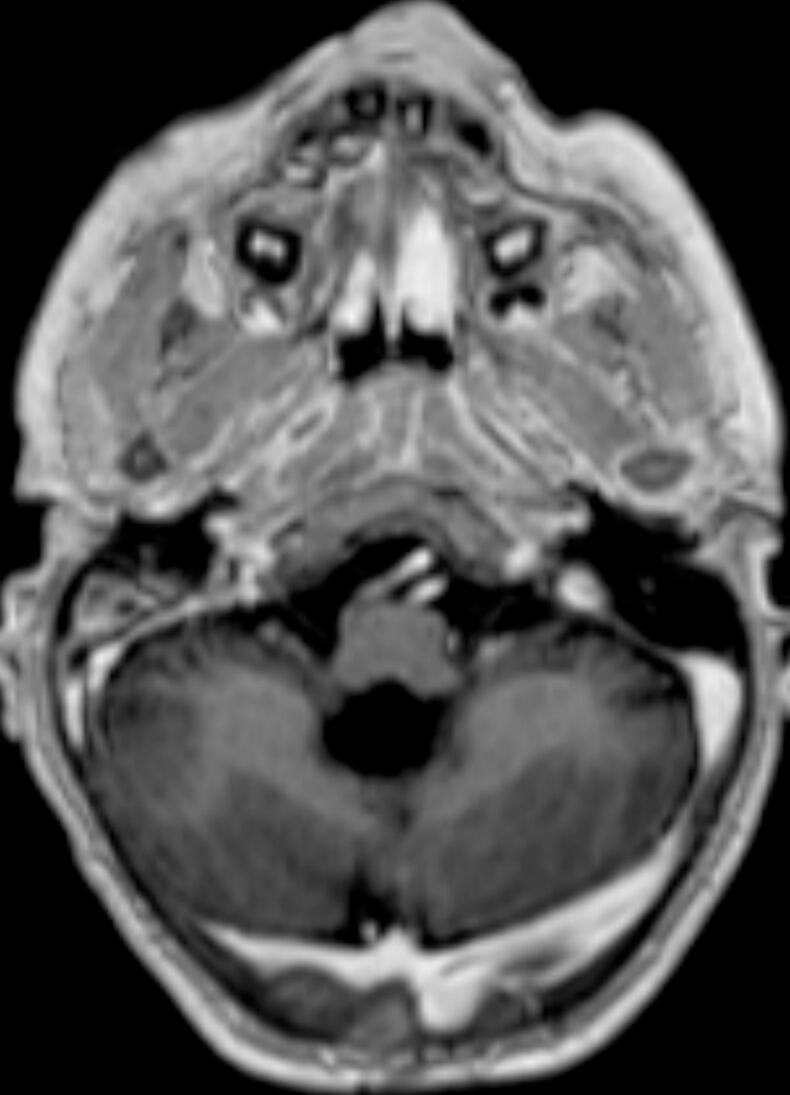
Axial cut of a T1 MRI seqquence done on Febraury 2025. There is no evidence of radiological.

The patient underwent craniotomy and surgical excision in January 2021. A post-procedure MRI revealed a residual enhancing lesion and the histopathological analysis confirmed the presence of an ATRT (Figs. 2 A, B, and C). ACNS0333 chemotherapy regimen was initiated. A second MRI on February 5, 2021, revealed an increase in the size of the enhancing residual lesion (Figs. 3 A, B, and C). A second craniotomy was done on April 16, 2021, with complete intraoperative resection. The patient was followed with interval MRI until February 2025 (Fig. 4, Fig. 5, Fig. 6, Fig. 7). The patient is symptom-free with no radiological evidence of recurrence.

## 3 Discussion

Notably, the age-dependent disparity in metastatic disease underscores the distinct pathological characteristics in infants, with all those under six months displaying metastatic involvement, compared to 35.3 % in older infants [[9], [10], [11], [12], [13], [14]]. The workup for primary lesions and metastasis was negative. No pathology was detected on a whole-body CT scan. All tumor markers were negative. The definitive diagnosis necessitates immunohistochemistry and genetic testing due to the limited representation of rhabdoid cells in the specimen. The commonality of inactivation of both copies of the integrase interfactor-1 (INI-1) gene and the expression of epithelial membrane antigen (EMA), vimentin, and smooth muscle actin (SMA) in rhabdoid cells further underlines the molecular intricacies of ATRT.

Histologically akin to medulloblastomas, ATRTs demonstrate an embryonic origin, featuring rhabdoid cells and a composite structure comprising neuroectodermal, ectodermal, and mesenchymal elements, classifying them as Grade 4 tumors according to the World Health Organization (WHO) criteria. The diagnostic process involves magnetic resonance imaging (MRI), revealing distinct radiological features such as hyperintensity on T2-weighted images and heterogeneous enhancement on T1 images with contrast, mirroring similarities with medulloblastomas. The imaging findings in this patient align with the literature. ATRTs typically present as mixed solid-cystic lesions, with hyperintensity on T2-weighted images and heterogeneous enhancement on T1 images with contrast. The MRI findings in this case showed a mixed solid-cystic lesion, which is consistent with the known radiological characteristics of ATRTs. While ATRTs and medulloblastomas share some imaging characteristics, such as hyperintensity on T2-weighted images and contrast enhancement on T1, ATRTs often exhibit more heterogeneous enhancement due to their mixed cellular composition. Medulloblastomas, on the other hand, are usually more homogeneously enhancing and less likely to be cystic. The imaging features in this patient suggest similarities but also highlight key differences.

Despite the preoperative differential diagnosis, surgery is a key component of treatment for both ATRTs and medulloblastomas. In both cases, maximal safe resection improves prognosis by reducing tumor burden before adjuvant therapy. Therefore, even if the diagnosis were medulloblastoma, surgery would still have been beneficial. However, the management would have differed primarily in the choice of adjuvant therapy. ATRTs require an aggressive multimodal approach, including intensive chemotherapy (e.g., ACNS0333 regimen) and often radiation, depending on the patient’s age. Medulloblastomas are typically managed with surgical resection followed by risk-stratified treatment, including craniospinal irradiation and chemotherapy, which varies depending on molecular subtypes. In this case, if the lesion had been a medulloblastoma, treatment might have included craniospinal irradiation earlier, given the high risk of CSF dissemination in medulloblastomas. However, since ATRTs are more aggressive, the chosen management approach for this patient was appropriate.

Despite advancements in therapeutic modalities, a standardized treatment protocol for ATRTs remains elusive, contributing to a particularly grim prognosis with a mean survival of 6–18 months. The challenges are further underscored by the observed recurrence at the primary tumor site and the occurrence of distant metastases in blood and cerebrospinal fluid (CSF), both major contributors to patient mortality. The discussion delves into the complexities of disease progression (PD), documented in 77 % of cases within a median of 160 days post-diagnosis. Moreover, a nuanced exploration of the literature reveals potential correlations between improved survival and various factors, including the absence of metastasis at diagnosis, early adjuvant radiotherapy and high-dose chemotherapy (HDCT), and diagnoses made after the year 2011, indicative of evolving therapeutic paradigms [[15], [16], [17], [18], [19], [20], [21]].

The summarized studies in Table 1 highlight the challenges and variability in ATRT presentation, treatment, and outcomes [[22], [23], [24]]. The Children’s Oncology Group (COG) ACNS0333 study, with 64 cases, underscores the aggressive nature of ATRT and the necessity of intensive chemotherapy and surgical intervention, reporting a 4-year overall survival (OS) of 54 % for infratentorial cases. Similarly, the case report by Paun et al. describes a posterior fossa ATRT managed with a two-stage surgical approach and chemotherapy, leading to a positive outcome. The case series in *Frontiers in Surgery further* emphasizes the importance of multimodal therapy in achieving a better prognosis. Compared to these studies, our case aligns with the literature in terms of presentation—manifesting with ataxia and gait disturbances—and radiological characteristics, showing a solid cystic lesion in the posterior fossa. However, despite an initial recurrence, our patient demonstrated long-term survival without recurrence, suggesting that early aggressive surgical intervention combined with ACNS0333-based chemotherapy may improve outcomes in select pediatric ATRT cases.

**Table 1:** Summary of selected studies reporting treatment approaches and outcomes in infants and young children with Atypical teratoid/rhabdoid tumors (ATRTs), highlighting tumor location, treatment modalities, and survival outcomes.

Study	Number of cases	Age range	Tumor location	Treatment	Outcome
Reddy et al., [22]	64	< 3 years	Infratentorial and Supratentorial	Surgery + Intensive Chemotherapy ± Radiation	4-year OS: 54 % (infratentorial), 35 % (supratentorial)
Paul et al. [23]	1	9 months	Posterior Fossa	Two-stage surgery + Chemotherapy	Positive outcome, survival beyond standard prognosis
Gou et al. [24]	12	16–30 months	Infratentorial and Supratentorial	Surgery + Chemotherapy ± Radiation	Highlighted multimodal therapy benefits

## 4 Conclusion

This case highlights the complexities of managing pediatric ATRT, emphasizing the importance of a multidisciplinary approach. The imaging findings were consistent with ATRTs, showing similarities to medulloblastomas but with distinct characteristics. Surgery played a crucial role in tumor control, and while management would have differed for medulloblastoma—particularly with earlier craniospinal irradiation—the aggressive multimodal approach used here was essential for ATRT. This case underscores the challenges in achieving complete tumor eradication and the necessity of close radiological and clinical follow-up to optimize patient outcomes.

## Consent

Consent was taken from the mother of the patient because he is a pediatric patient. The consent form is signed and available upon request.

## Ethical approval

The study was exempted from ethical approval as the information was reported anonymously.

## Disclaimer

This article was reported according to the SCARE criteria [25].

## Sources of funding

This study was not funded.

## Declaration of competing interest

The authors declare no conflict of interest.
